# Genetic and ElectroNic medIcal records to predict oUtcomeS in Heart Failure patients (GENIUS-HF) - design and rationale

**DOI:** 10.1186/1471-2261-14-32

**Published:** 2014-03-04

**Authors:** Luciana Gioli-Pereira, Sabrina Bernardez-Pereira, Fabiana Goulart Marcondes-Braga, Joceli Mabel Rocha Spina, Rafael Muniz Miranda da Silva, Noely Evangelista Ferreira, Fernando Bacal, Fábio Fernandes, Alfredo José Mansur, José Eduardo Krieger, Alexandre Costa Pereira

**Affiliations:** 1 Laboratory of Genetics and Molecular Cardiology, Heart Institute (InCor) of University of São Paulo Medical School, Avenue Dr. Enéas de Carvalho Aguiar, 44 Cerqueira César, São Paulo, SP 05403-000, Brazil; 2 Heart Institute (InCor) of University of São Paulo Medical School, São Paulo, Brazil; 3 Fluminense Federal University, Niteroi, Rio de Janeiro, Brazil

**Keywords:** Biobank, Genome-wide association, Electronic data, Cardiovascular outcomes

## Abstract

**Background:**

Studies adopting electronic medical records and genomic information are becoming widespread. Through this new modality in research, it is possible to study how genetic variants influence susceptibility towards chronic conditions and can improve patient care.

Our aim is to develop a biobank with 2,000 heart failure patients treated in a tertiary cardiology hospital containing electronic medical records data and biologic samples for performing genome-wide association studies for validation and development of medical decision routines aimed at helping the clinical management of patients.

**Methods/Design:**

Patients between 18 and 80 years old with heart failure diagnosis of different etiologies and left ventricular ejection fraction ≤ 50% in the past 2 years will be eligible for enrollment on the cohort. After consent, patients will be submitted to clinical baseline, echocardiography, cardiograph impedance and biochemical evaluation. Study data will be collected and managed using Research Electronic Data Capture tools. The follow up will take place every 6 months to assess cardiovascular outcomes (all-cause mortality, cardiovascular mortality, hospitalization for worsening heart failure and current medication use). Initial analytical strategy will focus on the establishment of the accuracy of electronic medical records extraction protocols for main predictor factors of morbidity and mortality in heart failure.

**Discussion:**

Building a biobank with biologic samples and clinical data of 2,000 heart failure patients we will perform genome-wide association studies. By this way, we pretend to study how genetic variants influence susceptibility towards chronic conditions. Besides, it will be created a working group focused on the development and implementation of algorithms for validation and application of medical routines using the electronic medical records of the Heart Institute (InCor - HCFMUSP).

**Trial registration:**

Current Controlled Trials NTC02043431.

## Background

Heart failure is an important cause of morbidity and mortality worldwide and in Latin America, the risk factors and epidemiology have peculiarities that are still unclear. Recently, Bocchi et cols discussed the similarities in risk factors compared to developed countries highlighting the prevalence of others causes like Chagas’disease, rheumatic fever and lower total expenditure on health per capita [1].

The mapping of the human genome has enabled new exploration on how genetic variation contributes to health and disease [2]. Technological advances in the understanding of disease processes are presented as new opportunities to improve healthcare through management of individual patients. In this context, the adoption of electronic medical records (EMR), used by clinicians to document clinical care, is becoming widespread and recent studies demonstrate that they can be effectively employed for genetic studies using the informational and biological ‘by-products’ of healthcare delivery while maintaining patient privacy [3].

This new approach in research leads on the Personalized Medicine Research Project, a population-based biobank that involves discussions about community consultation and other legal aspects [4]. Actually, efforts to improve patients’ understanding of their own medical treatments or research in which they are involved are progressing, especially with regard to informed consent procedures [5].

Recently, the National Human Genome Research Institute (NHGRI) called on researchers to determine how genetic variants influence susceptibility towards chronic conditions such as diabetes, Alzheimer’s disease, and cardiovascular disease, in order to ultimately improve patient care. For this purpose, the eMERGE (electronic MEdical Records and GEnomics) Network – an NHGRI-supported consortium of five institutions – was created to explore the utility of DNA repositories coupled to EMR systems for advancing discovery in genome science [6].

Allied to this new modality in research, the adoption of an online database allows easy access and processing of information collected in a safe and efficient way. The Research electronic data capture (REDCap) is a novel workflow methodology and software solution designed for rapid development and deployment of electronic data capture tools to support clinical and translational research. It was initially developed and deployed at Vanderbilt University, but now has collaborative support from a wide consortium of domestic and international partners [7].

It is clear that new strategies are essential for prevention and treatment of chronic diseases like heart failure. Conditions described in Latin America that has peculiar epidemiology compared to other countries, need further studies. In this scenario, our aim is to develop a biobank containing samples of 2,000 patients with heart failure diagnosis treated in a tertiary cardiology hospital containing electronic medical record and genetic data in genome-wide scale for performing genetic association studies for validation and development of medical decision routines that help the clinical management of these patients.

## Methods/Design

### Research design

Subjects with systolic heart failure, sequentially included, will be submitted to clinical baseline, echocardiography, cardiograph impedance and biochemical evaluation, which include blood, urine and exhaled breath acetone samples. It will allow the authors to best define the heart failure syndrome, its etiology and its hemodynamic profile. All patients included will also be submitted to clinical follow-up every 6-month for 3 years to assess cardiovascular outcomes: all-cause mortality, cardiovascular mortality, hospitalization for worsening heart failure, current medication use and heart transplantation. The diagnosis of heart failure will be made according to previously published criteria [8]. To define the appropriate etiology of cardiomyopathy, the authors will follow previous guidelines [9,10].

### Patients selection

The individuals invited to our study will be consecutively selected from the Heart Institute - Clinical Hospital- University of São Paulo Medical School (InCor - HCFMUSP) on their outpatient admission, starting at January 2011.

### Ethics

The study protocol was approved by the Ethics Committee for Medical Research on Human Beings of the Clinical Hospital from University of São Paulo Medical School (SDC 2368/03/162). Signed informed consent will be obtained from all participants.

### Inclusion criteria

Patients between 18 and 80 years old with heart failure diagnosis of different etiologies and left ventricular ejection fraction ≤ 50% on two-dimensional transthoracic Doppler echocardiography performed in the past 2 years, who are admitted to the Heart Institute from January 2011, will be eligible for enrollment on the cohort. Specifically, patients with cardiomyopathy due to valvular heart disease enrolled in the study will be those with severe left ventricular dysfunction to the point that they would not be eligible for valve repair or replacement, but rather candidates for heart transplantation.

### Exclusion criteria

Patients with impaired cognition due to advanced dementia syndrome or severe psychiatric disorder, without telephone access or that refuse to participate will be excluded.

### Left ventricular function assessment

During echocardiography, transthoracic images will be obtained with the patient in the left lateral position using Acuson Sequoia 512 (Siemens). The image acquisitions will be standardized in the four windows: parasternal long and short axis, and apical 4 - and 2-chambers. The evaluation of the left ventricular (LV) function will be performed by the echocardiography staff in a blinded way in relation to the protocol and will be conducted according to previously standard recommendations. Left ventricular systolic and diastolic volumes and ejection fraction will be derived from biplane apical views using the modified Simpson’s rule algorithm. Diastolic dysfunction will be determined using Doppler measurements of mitral inflow and Doppler tissue imaging (DTI) of the mitral annulus.

### Biobanking

All enrolled patients will have fasting serum, exhaled breath acetone, plasma and urine samples collected and stored at -80°C for further analysis.

### Genomic DNA protocol

Genomic DNA will be extracted from peripheral leucocytes after a standard salting out protocol [11]. DNA quality and integrity will be checked in all samples after extraction protocol.

### Whole-blood RNA collection

The enrolled patients will have whole-blood RNA collected in fasting state and stored in PAXGene tubes for further analysis according to standard protocol [12].

### Exhaled breath acetone (EBA)

The exhaled breath acetone has been described as a good biomarker of the diagnosis of heart failure as previously described [13] and will be used associated to BNP (B-type natriuretic peptide) and clinical criteria. After collection of blood samples, patients will have standard diet and will be submitted to the exhaled breath acetone collection by using a simple and portable device. After collection, the liquid phase of the sample will be transferred from the device to a capped vial and stored in a freezer at -80°C until acetone analysis.

### Studied variables

Enrolled patients will have the opportunity to contribute to genetic associations regarding a number of demographic, clinical and follow-up information. All included individuals will have data on sex, ethnicity, age, duration of symptoms, etiology of heart failure, history of diabetes mellitus, arterial hypertension, smoking, drugs and morbidity outcomes. Anthropometric data like body mass index, heart rate, blood pressure, cardiac rhythm, cardiac dimensions on echocardiography and left ventricle ejection fraction (both baseline and sequential in one year intervals). We will perform serum analyses (sodium, potassium, total cholesterol, triglycerides, HDL-cholesterol, LDL-cholesterol, VLDL-cholesterol, glucose, creatinine, urea, B-type natriuretic peptide), exhaled breath acetone dosage and urine analyze. Follow-up will be assessed every 6 months to investigate clinical outcomes as cited above. In addition, the mortality database of São Paulo City Authority will also be periodically scrutinized to discover patient deaths (ProAim-Programa de Aprimoramento de Informações de Mortalidade do Municipio de São Paulo). Follow-up will be carried for all individuals until a 3-year period.

### Electronic data collection (REDCap)

Study data will be collected and managed using REDCap electronic data capture tools hosted at the Clinical Hospital from University of São Paulo Medical School. REDCap (Research Electronic Data Capture) is a secure, web-based application designed to support data capture for research studies, providing: 1) an intuitive interface for validated data entry; 2) audit trails for tracking data manipulation and export procedures; 3) automated export procedures for seamless data downloads to common statistical packages; and 4) procedures for importing data from external sources [7].

### Cardiograph impedance

Hemodynamic data will be obtained noninvasively using a monitor CardioScreen 2000 (Medis, Germany) approved for marketing by U.S. Food and Drug Administration (FDA). This device measures the thoracic bioimpedance using the following technique: four dual sensors (each sensor consists of two electrodes) are placed on the patient, on opposite sides of the neck, at a level between the ears and shoulders and on both sides of the chest in the mid-axillary line at the level of the xiphoid process. The electrodes transmit a current of low amplitude and high frequency (2.5 mA and 70 kHz), and detect changes in voltage chest. These alterations in voltage are used to calculate the impedance (Z). The following parameters will be assessed: Cardiac index (CI), Stroke Volume Indexed (SVI), Mean Arterial Pressure (MAP), systemic vascular resistance index (SVRI), Thoracic Fluid Content Indexed (CLTI), velocity index, acceleration index, pre-ejection period, LV ejection time, and systolic time ratio (inversely proportional to LV function; pre-ejection period divided by LV ejection time).

### Genome-wide association studies (GWAS)

The genomic DNA samples will be processed and genotyped in the Affymetrix biobank platform for around 800.000 polymorphic markers. For quality control, will be deleted SNPs with genotypes not identified in > 20% of the samples, SNPs with allele frequency rarer (MAF) <1%, or results to Hardy-Weinberg test <0.001. Further, after exclusion of SNPs with quality problems in genotyping, individuals will be analyzed for consistency of genotyping. Individuals with genotyping rate <90% will be excluded.

### Development of diagnostic algorithms based on electronic medical records

The Heart Institute (InCor - HCFMUSP) had developed and used an in-house system of electronic medical records in the last 2 decades. This information system has medical data that need to be validated for use in systematic clinical research. Using these medical records we pretend to create algorithms for data extraction that enable selection and classification of individuals, or the extraction of continuous variables of medical interest that can be used in research protocols or development of medical decision tools. In summary, we intent to evaluate the validity and specificity of this data collection system and apply it in genetic association studies as well as medical routines of the Institute.

### Statistical analysis

Data will be analyzed using SPSS for Windows version 13.0 (SPSS Inc) statistical software.

## Discussion

We expect to develop a biobank containing samples and clinical data of 2,000 patients with heart failure diagnosis for performing genetic association studies. Besides, we aim to create a working group focused on the development and implementation of algorithms for validation and application of medical decision routines using our electronic medical record system in order to help the clinical management of patients at the Heart Institute (InCor - HCFMUSP). Furthermore, the project will focus on ethical, legal, political and social impact of this new reality, such as privacy, confidentiality and possible interactions with the community. In this sense, we intend to identify sources of concern among participants regarding current approaches to obtaining informed consent for storage and use of genetic material in clinical research.

In this way, with the appropriate use of genetic data in genome-wide scale associated to electronic medical information, we expect to accelerate both the discovery of genetic factors associated with complex diseases such as heart failure and create medical decision routines that help the clinical management of patients in a tertiary cardiology hospital.

## Abbreviations

EMR: Electronic medical records; NHGRI: National Human Genome Research Institute; eMERGE: electronic MEdical records and GEnomics; REDCap: Research electronic data capture; LV: Left ventricular; DTI: Doppler tissue imaging; EBA: Exhaled breath acetone; BNP: B-type natriuretic peptide; FDA: Food and Drug Administration; Z: Impedance; CI: Cardiac index; SVI: Stroke volume indexed; MAP: Mean arterial pressure; SVRI: Systemic vascular resistance index; TFCI: Thoracic fluid content indexed; GWAS: Genome-Wide Association Studies.

## Competing interests

The authors declare that they have no potential conflicts of interest regarding the present publication.

## Authors’ contributions

LGP performed the acquisition of data, drafted the manuscript. SBP performed the acquisition of data, helped to draft the manuscript. FGMB performed the acquisition of data, helped to draft the manuscript. JMRS performed the collection of data, carried out part of the molecular genetic studies. RMMS performed the collection of data. NEF carried out part of the molecular genetic studies. FB helped in the patients recruitment. FF helped in the patients recruitment. AJM participated in study design and helped in the patients recruitment. JEK conceived the study, and participated in its design. ACP conceived of the study, and participated in its design and coordination and helped to draft the manuscript. All authors read and approved the final manuscript.

## Pre-publication history

The pre-publication history for this paper can be accessed here:

http://www.biomedcentral.com/1471-2261/14/32/prepub
